# A revised acceleration rate from the altimetry-derived global mean sea level record

**DOI:** 10.1038/s41598-019-47340-z

**Published:** 2019-07-29

**Authors:** Marcel Kleinherenbrink, Riccardo Riva, Remko Scharroo

**Affiliations:** 1 Geoscience and Remote Sensing, Delft University of Technology, Delft, Netherlands; 2 Astrodynamics and Space Missions, Delft University of Technology, Delft, Netherlands; 3 European Organisation for the Exploitation of Meteorological Satellites, Darmstadt, Germany

**Keywords:** Physical oceanography, Physical oceanography

## Abstract

Satellite radar altimetry has been providing estimates of global mean sea level (GMSL) since 1992. The early TOPEX record originates from two identical instruments, which requires the estimation of an intermission bias and careful handling of the problematic first part of the record. Calibration of TOPEX is crucial to obtain a continuous and consistent record, which is needed to quantify any recent acceleration. We propose a novel approach to calibrate TOPEX altimeter data using sea surface height differences at crossovers of TOPEX and ERS. Tide gauges are only used to determine a drift in one of the two datasets. We provide a new and more accurate estimate of the intra-mission bias, which leads to a much reduced GMSL acceleration over the whole record. Hence, the conundrum of an uncertain GMSL acceleration from altimetry is still unsolved, in spite of recent opposite claims, and in contrast to the expected effect of ocean warming and continental freshwater fluxes.

## Introduction

Data from the satellite radar altimetry missions TOPEX/Poseidon (1992–2002), Jason-1 (2002–2008) and Jason-2 (2008–2016) need to be merged in order to construct continuous time series of GMSL. This requires to align the timeseries by removing biases between the observations of two consecutive missions, which is usually based on a period of several months, when they fly in a tandem constellation, after which the oldest satellite mission is moved to another orbit and eventually decomissioned. An exception is represented by the TOPEX/Poseidon mission, hosting the TOPEX instrument that was actually constituted by two identical devices (called side A and side B). The mission started in 1992 by operating side A, but it was switched to its redundant B-side in 1999, due to technical issues^1^.

In order to ensure stability of the GMSL record, a selection of tide gauges is used for an independent validation^2,3^. The tide-gauge comparison is also used to remove an intramission bias between TOPEX-A and TOPEX-B, since there are no overlapping observations during the instrumental change. Over the TOPEX-A period, the tide-gauge validation revealed a U-shaped drift in TOPEX sea surface heights^3^, which has been related to the degradation of the Point Target Response (PTR) of the instrument^4^. The PTR describes the expected power of reflected signals and it is assumed to remain constant over time. Hence, its degradation will affect the whole processing chain: the determination of geophysical parameters like sea surface height, wind speed and Significant Wave Height (SWH), the instrumental calibration for internal path delays (cal-1)^4^, and the estimated Sea-State Bias (SSB)^5^. In particular, the magnitude of the U-shaped drift is large enough to prevent detection of an acceleration in GMSL.

Three independent solutions to remove the drift have been proposed. Firstly, Watson *et al*.^6^ calibrated the TOPEX altimeter with tide gauges by removing two separate secular drifts from TOPEX-A and TOPEX-B time series, as well as an intramission bias. Watson *et al*.^6^ also calibrated the Jason altimeters by removing secular drifts. This yielded a positive, but statistically insignificant, acceleration in GMSL of 0.041 ± 0.058 mm yr^−2^. Chen *et al*.^7^ and Dieng *et al*.^8^ showed that the sum of sea level contributors better matched altimetry-derived GMSL after the calibration of Watson *et al*.^6^. Secondly, Beckley *et al*.^4^ showed that the U-shaped drift in TOPEX-A matched the shape of the cal-1 correction and removed cal-1 from the TOPEX time series. They did not apply any intramission bias and found a statistically significant acceleration in GMSL of 0.051 ± 0.020 mm yr^−2^, which is based on altimeter data alone. Nerem *et al*.^9^ used this approach, but applied an intramission bias and corrected for several interannual dynamics to estimate a statistically significant climate-related acceleration in GMSL of 0.084 ± 0.025 mm yr^−2^. A similar acceleration (0.10 mm/yr^−2^) was found by Cazenave *et al*.^10^ based on the mean of three drifts computed using the Poseidon altimeter, a tide-gauge comparison and the sea level budget. Thirdly, Beckley *et al*.^4^ used TOPEX data, which was processed accounting for a changing PTR^11^ and found a statistically equal acceleration (0.061 ± 0.025 mm yr^−2^) as the second approach without applying an intramission bias. The last approach seems more sound, because it does not rely on a combination of several ranges, SSB and cal-1 corrections. However, the first 1.5 years of the timeseries had to be omitted due to data processing issues.

We challenge these solutions by means of a crossover analysis between TOPEX an ERS-1&2 and we derive a revised acceleration rate, whose point estimate is much reduced in magnitude with respect to those previous studies. The crossover analysis shows that not applying cal-1 is justified and removes the U-shaped drift in TOPEX-A. However, the removal of cal-1 from the whole TOPEX record leads to an intramission bias with an amplitude that depends on the applied SSB correction. Additionally, this approach reveals the presence of a drift of −1.1 ± 0.3 mm yr^−1^ between the TOPEX sea surface heights and those of ERS-1&2. An alternative approach consists in removing cal-1 from TOPEX-A alone, which yields a negligible intramission bias in case the Gaspar *et al*.^12^ SSB correction is applied. Also in this case, a drift between TOPEX and ERS of −1.3 ± 0.1 mm yr^−1^ is found, with is statistically equivalent to the previous value.

Based on four different weighting methods used in a tide-gauge comparison it is determined that TOPEX is drifting and not ERS. Therefore, we suggest to calibrate the TOPEX GMSL record with the crossover of ERS1&2 after the removal of cal-1. The calibration reduces the observed acceleration in GMSL, so that it becomes statistically equivalent to zero at the 95%-confidence level. The result of the calibration is independent of the applied SSB correction. Note that we do not claim an acceleration in GMSL is absent, rather that a positive acceleration in GMSL based on the altimetry-derived record is likely (>66% confidence) instead of virtually certain as suggested by other studies.

## Crossover Analysis

**Figure 1:**
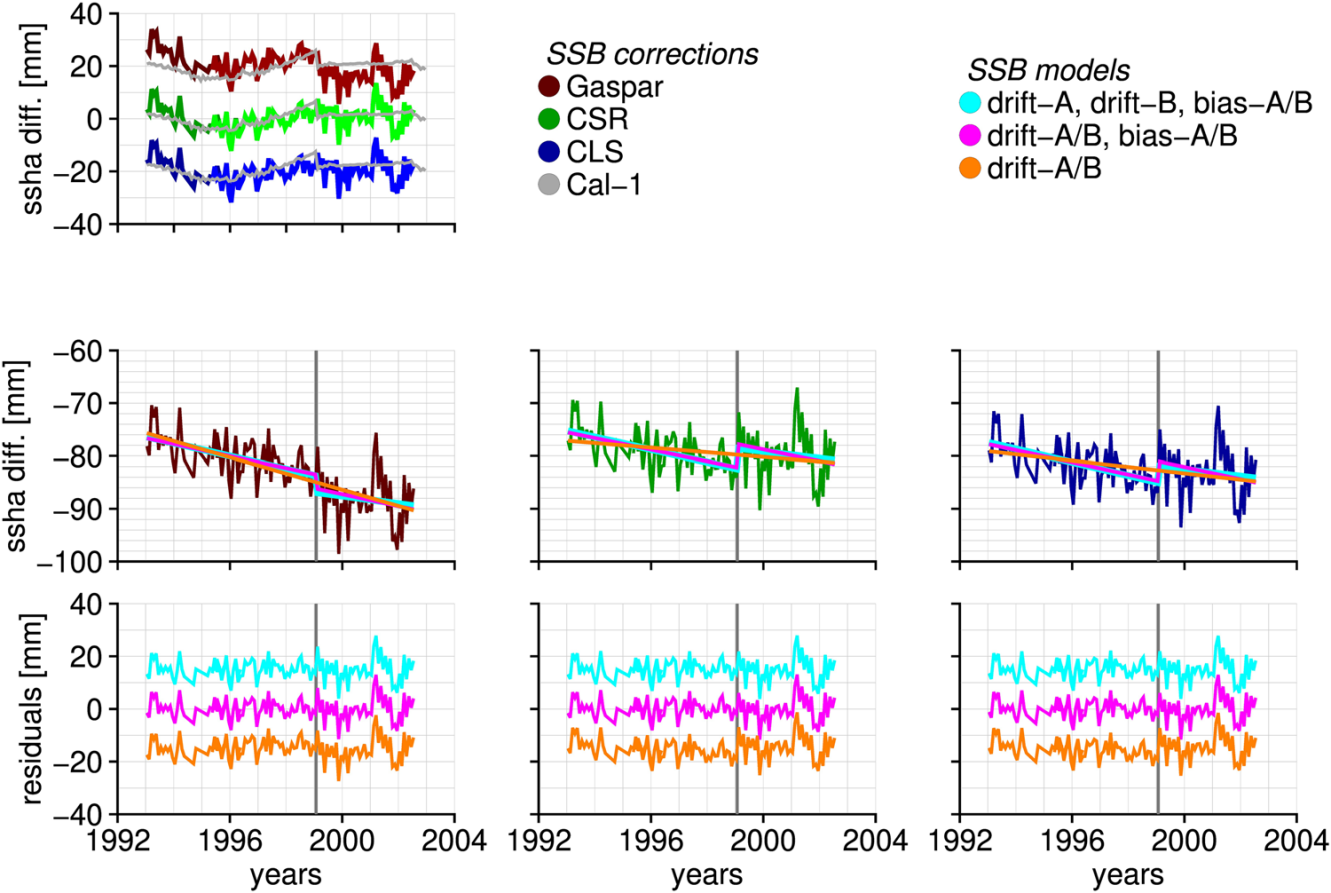
Crossover differences between TOPEX and ERS-1&2 for three SSB correction. The top figure shows the difference time series for cal-1 applied. The middle figures show the three model fits after cal-1 is removed. The bottom figures show the residuals. Arbitrary offsets are added for visibility.

Sea surface height differences at crossovers between TOPEX and ERS-1 (1993–1996) and TOPEX and ERS-2 (1995–2002) are globally averaged to create the monthly time series shown in Fig. 1. The three time series correspond to two parametric SSB corrections, Gaspar *et al*.^12^ (short: Gaspar) and Chambers *et al*.^13^ (short: CSR), and a non-parametric one, Labroue *et al*.^14^ (short: CLS). More details on the SSB corrections are provided in the Supplementary material. A non-linear curve that resembles the cal-1 correction time series is present independent of the applied SSB correction, which was also observed in tide-gauge comparisons^3,4,6^. As Beckley *et al*.^4^ suggested, the removal of cal-1 appears to be justified, because it removes the U-shaped drift in the TOPEX-A record.

After removing cal-1 the residual TOPEX-ERS timeseries exhibits a drift and a discontinuity at the moment of the TOPEX-A/B transition in January 1999 (Fig. 1, middle three panels). We fit three models to the time series: 1) a single drift through TOPEX-A/B, 2) a single drift through TOPEX-A/B and an intramission bias and 3) separate drifts for TOPEX-A&B and an intramission bias. The score in Table 1 is based on a F-test (Supplementary material) and indicates that, for the CSR and CLS SSB corrections, fitting models 2 and 3 significantly reduces the residuals with respect to fitting model 1. The TOPEX-A/B drift for the Gaspar SSB correction is significantly different than the other two. Also note that the TOPEX-A&B drifts are statistically equivalent within the 95% confidence interval, which suggests that fitting a single drift through the whole time series is enough. Independent of the SSB correction, we therefore suggest to fit model 2, because it leads to statistically equivalent drifts between TOPEX and ERS1&2.

**Table 1:** Estimated drifts and biases from crossovers of TOPEX-ERS1&2. The F-scores are computed with respect to model 1, with high scores indicating a significant improvement. Bold numbers indicate no significant improvement with respect to model 1. *For model 2 the 5% significance level is 3.9, while for model 3 it is 3.1.

SSB	Cal-1	Model	Drift A [mm yr^−1^]	Drift B [mm yr^−1^]	Bias A/B [mm]	F-score*
Gaspar	no	1	−1.53 ± 0.14		—	—
CSR	no	1	−0.44 ± 0.14		—	—
CLS	no	1	−0.59 ± 0.14		—	—
Gaspar	B	1	−1.25 ± 0.13		—	—
Gaspar	no	2	−1.13 ± 0.25		−2.7 ± 1.4	**3.6**
CSR	no	2	−1.10 ± 0.25		4.4 ± 1.4	10.0
CLS	no	2	−1.15 ± 0.25		3.7 ± 1.4	7.1
Gaspar	B	2	−1.07 ± 0.24		−1.2 ± 1.4	**0.7**
Gaspar	no	3	−1.22 ± 0.27	−0.71 ± 0.61	−3.2 ± 1.5	**2.1**
CSR	no	3	−1.19 ± 0.27	−0.64 ± 0.60	3.9 ± 1.5	5.3
CLS	no	3	−1.25 ± 0.27	−0.64 ± 0.60	3.1 ± 1.5	4.0
Gaspar	B	3	−1.22 ± 0.27	−0.34 ± 0.60	2.1 ± 1.5	**1.3**

In contrast to Beckley *et al*.^4^, it is also possible to treat TOPEX-A&B as two separate altimeters and therefore only apply cal-1 to TOPEX-B. If the Gaspar SSB correction is applied, the F-scores show that the application of models 2 and 3 does not lead to an improved fit with respect to model 1. Therefore no intramission bias has to be estimated. The resulting drift between TOPEX and ERS1&2 is statistically equivalent to those found for model 2. Note that the drift estimate is relative to ERS1&2 and that a tide-gauge comparison is required to verify whether the TOPEX or ERS sea surface heights are drifting.

## Tide-Gauge Comparison

Validation and calibration of TOPEX has often been done by means of tide gauges and it usually implies that separate drifts are estimated for TOPEX-A&B, together with an intramission bias. Tide-gauge comparisons are problematic, in particular because 1) the tide gauges are heterogenously distributed and 2) the differentiation of altimetry and tide-gauge records does not completely remove ocean signals. The heterogenous distribution of tide gauges leads to the under- and overweighting of certain regions, depending on the averaging method applied. A crossover analysis with ERS-1&2 shows that the TOPEX intramission bias and the TOPEX-B drift are geographically varying (Supplementary material). Therefore the results of the tide-gauge comparison are likely biased. Kleinherenbrink *et al*.^15^ showed that ENSO signals are detectable in differenced altimetry - tide-gauge time series at Winter Harbour at the west coast of Canada. Since an El Niño (1997–1998) and a La Niña (1999–2000) events occurred around the time of the TOPEX-A/B switch and many tide gauges are located in the Pacific, both the estimated drifts and the intramission bias might be biased.

To investigate these issues, four methods are applied to estimate the mean drifts and the intramission bias for TOPEX-A&B (Sect. 5). Application of model 3, as described in Sect. 2, indicates that the TOPEX-B drift is difficult to constrain and varies 2–3 mm yr^−1^ (Supplementary material). Application of models 2–3 also gives intramission bias differences of approximately 3 mm and approximately 5 mm lower than those obtained from the crossovers (Supplementary material). We argue that the time series of TOPEX-A&B are too short and the tide gauges are too heterogenously spaced to be able to estimate an accurate intramission bias.

**Figure 2:**
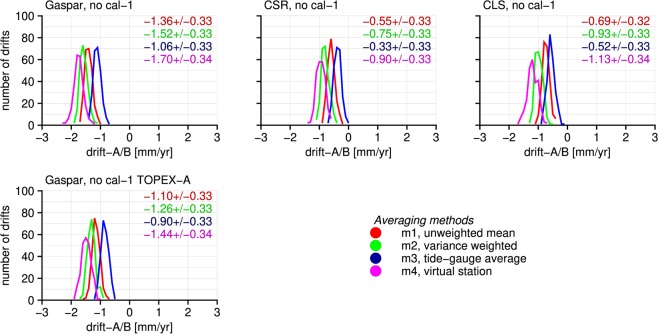
Histograms of TOPEX-A/B drifts estimated from a tide-gauge comparison when no intramission is estimated. The drifts are statistically equivalent to the drifts estimated using crossovers, indicating that TOPEX is drifting and not ERS-1&2.

Figure 2 therefore only shows the histograms of TOPEX-A/B drifts obtained with the application of model 1. With all four averaging methods TOPEX-A/B drifts are estimated that are statistically equivalent to each other and also to those obtained with the crossovers, independent from the applied SSB correction. Note that for the CLS and CSR SSB corrections, these results are statistically equal to the −0.45 mm yr^−1^ drift found by Beckley *et al*.^4^. However, model 1 is not the best fit with the crossover time series when the CSR or CLS SSB correction were applied and the F-score indicated that Gaspar time series might require the estimation of an intramission bias as well. According to the crossover analysis, no intramission bias has to be estimated when cal-1 is applied to TOPEX-B only in combinationn with the Gaspar SSB correction. Also these tide-gauge-derived drifts are equivalent to those estimated with the crossovers. This shows that the TOPEX measurements are drifting and not the ERS1&2 measurements.

Therefore it is proposed to calibrate the GMSL time series of TOPEX with the drifts and intramission biases estimated from the crossover analysis. If any of the three SSB corrections is applied and cal-1 is not applied to the TOPEX-A&B time series, then a drift and intramission bias should be removed. Or, when the Gaspar SSB correction is applied, while cal-1 is removed from TOPEX-A, then only a drift should be removed.

## Accelerations in Global Mean Sea Level

The GMSL time series span 1993.0–2016.6 and are constructed from TOPEX, Jason-1 and Jason-2 data. A quadratic term is regressed together with a secular trend, annual and semi-annual cycles. The estimated quadratic coefficient is multiplied by a factor of two to obtain the acceleration.

**Figure 3:**
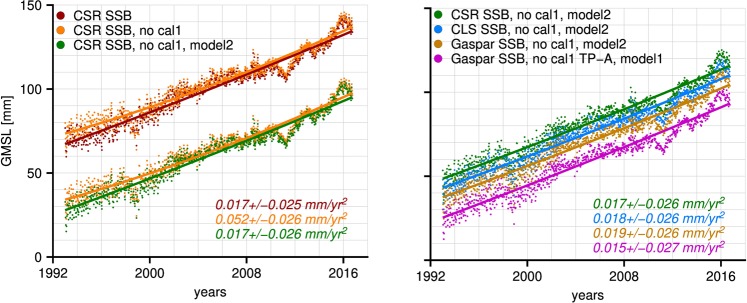
Residuals time series after removing the seasonal cycles for crossover time series based on various combinations of SSB, cal-1 and crossover model corrections. The solid lines represent the estimated quadratic curves and the listed values the corresponding accelerations and their 95% confidence interval.

The left panel in Fig. 3 shows the time series of the CSR SSB-based GMSL record before and after removing cal-1, and after the calibration with ERS crossovers without the seasonal cycles. Removing cal-1 and/or model 2 from the crossover calibration has an insignificant effect on the estimated secular trend (Supplementary material). If cal-1 is removed from TOPEX a significant acceleration is estimated, which is equivalent to the results obtained by Beckley *et al*.^4^. The acceleration becomes smaller and statistically insignificant again after calibration with model 2.

The right panel of Fig. 3 shows four ERS-calibrated time series. For three of them cal-1 is not applied, after which they are calibrated with model 2, while for the remaining one cal-1 is applied to TOPEX-B only and calibrated with model 1. The results show that the suggested calibration procedures lead to statistically equal results, with an insignificant acceleration in GMSL. If corrections for the effect of the Pinutabo eruption and ENSO were taken into account^9,16^, the acceleration could decrease even further. As a consequence, it is likely that a climate-driven acceleration is present in the altimetry-derived GMSL time series, but it is not certain.

Note that the inability to state that an acceleration is present with certainty using satellite radar altimetry does not imply there is no acceleration at all. Its estimated value in this study is actually in line with the results of the 20^*th*^-century tide-gauge-based GMSL reconstruction by Dangendorf *et al*.^17^, notably 0.018 ± 0.016 mm yr^−2^. The uncertainties in the altimetry-derived estimate, however, cause the same acceleration to become statistically equivalent to zero at a 95%-confidence level. It should be stressed that, based on the decadal behavior of GMSL as shown in the same study, we need to be careful with comparing accelerations from records of different length.

## Methods

### Altimetry processing

The TOPEX and ERS1&2 data are obtained from the Radar Altimetry Database System (RADS)^18^. For the three missions we fixed all geophysical corrections as listed in the supplementary material, except for the TOPEX SSB correction. A build-in tool of RADS is used to find crossovers between TOPEX and ERS. Sea surface height anomalies with absolute values larger than 1 meter are removed from the analysis as well as observations within 200 km from the coast. Between ERS1&2 a latitude-dependent intermission bias is removed as in Ablain *et al*.^19^. The crossovers differences are averaged per 1-degree latitude band and then weighted by the corresponding sea surface area^20^ to obtain globally-averaged monthly TOPEX-ERS differenced time series.

### Tide-gauge comparison

For the ALT-TG comparison we modified the procedure of Watson *et al*.^6^. The hourly research quality tide gauge records (rqds) from the UHDSLC^21^ are used. Only records spanning the full TOPEX record (1993–2002.5) are considered. Those within a radius of 1000 km from a 7.5 moment-magnitude or larger earthquake event during the same period are removed from the analysis.

Within a range of 220 km around the tide gauge Control Points (CP) are set, at which altimetry measurements are stacked at every 6 km in the along-track direction. Observations closer than 30 km from the coast are omitted to avoid land signals contaminating the altimeter waveforms. The dynamic atmosphere, ocean tide, load tide and pole tide correction from RADS are not applied to the altimeter data. Instead, after differencing with the tide-gauge measurements, the solid Earth part of the pole tide is removed following the IERS2010 conventions. At every control point, the tide-gauge measurements are cubicly interpolated in time to the altimetry epochs and consecutively subtracted from the altimetry measurements. Differences larger than 1 meter are removed from the analysis. A CP is kept for further analysis, if at least 250 differenced measurements pass the criteria.

Vertical land motion is estimated at the tide gauges using either nearby Global Navigation Satellite System (GNSS) receiver or the sum of modeled GIA and present-day mass redistribution trends. The GNSS trends are extracted from the extensive NGL database. These trends are estimated with the robust MIDAS estimator^22^. The considered GNSS trends have an uncertainty better than 1 mm yr^−1^. If multiple trends meet the requirements within 50 km from the tide gauge the median of is taken. If no GNSS trends meet the requirements, the ICE-6G_C VM5a GIA model^23^ is used together with modelled vertical land motion due to present-day mass redistribution^24,25^. Since the GNSS trends are not computed over the same time span as TOPEX, non-linear behavior due to present-day mass redistribution causes a small discrepancy. Therefore, the modeled vertical land motion is also used to correct GNSS trends^24^.

Global averaging of the CPs is done in four ways. In the first method all CP drifts and intramission biases are unweigthedly averaged. The second methods uses the variance of the residuals as weight for the CPs. In the third method the drifts and intramission biases are first averaged per tide gauge and the result is averaged globally. The last method averages biases and drifts per tide gauge and then applies a virtual station method^26^ so that nearby station are combined into a single station. At the point no stations are in the vicinity of 500 km of each other the drifts and biases are globally averaged.

## Supplementary information

Supplement to: A revised acceleration rate from the altimetry-derived global mean sea level record
