# Supplementary material for: A revised acceleration rate from the altimetry-derived global mean sea level record
Source: Sci Rep. 2019 Jul 29;9:10908. doi: 10.1038/s41598-019-47340-z (PMC6662663; doi:10.1038/s41598-019-47340-z)
Supplement: Supplementary file 1 — Supplement to: A revised acceleration rate from the altimetry-derived global mean sea level record [file 41598_2019_47340_MOESM1_ESM.pdf]

Supplement to: A revised acceleration rate from the altimetry-derived global mean sea level record

Marcel Kleinherenbrink[1,2], Riccardo Riva[1], and Remko Scharroo[3]

[1]Geoscience and Remote Sensing, Delft University of Technology
[2]Astrodynamics and Space Missions, Delft University of Technology
[3]European Organisation for the Exploitation of Meteorological Satellites

June 25, 2019

1 Satellite radar altimetry data

The satellite radar altimetry data for TOPEX and ERS-1&2 are obtained from the Radar Altimetry Database System (RADS)[1]. The applied geophysical corrections are listed in Table 1. The orbits solutions used are computed as part of the Sea Level project of the Climate Change Initiative (SLCCI)[10]. Note that the pole tide from *Desai et al.*[2] is used in the crossover analysis, which contains the variations with respect to a linear mean pole. In the tide-gauge comparison, we use the pole tide as described in the IERS2010 conventions[3], which account for a non-linear mean pole. Mean polar motion is implicity taken into account in a Vertical Land Motion (VLM) correction for the tide gauges (Sect. 3). Furthermore, the tides and dynamic atmosphere correction are not applied for the tide-gauge comparison. The major tidal harmonics are regressed as part of the TOPEX drift estimation (Sect. 3).

2 Crossover analysis

Weighted means of the TOPEX-ERS-1&2 crossovers are computed for 34 and 87 months, respectively. Fig. 1 shows the crossover time series for the three applied SSB corrections. Indepent of the SSB correction, the time series exhibit a clear U-shaped curve over the TOPEX-A period (1993-1999). Whereas the

transition between TOPEX-A&B appears to be continuous for the CSR and CLS SSB correction, there is an observable discontinuity for the Gaspar-corrected time series. Note that estimating a intramission bias from these time series is non-trivial, since it strongly depends on the number of months before and after the TOPEX-A/B transition that are used in a model.

Table 1: List of geophysical corrections used for ERS-1&2 and TOPEX. *Not applied in the comparison with tide gauges. **Only the solid Earth part of the pole tide is corrected for in the comparison with tide gauges, following the IERS2010 conventions.

<table>
<tr><th>Correction</th><th>TOPEX</th><th>ERS-1&2</th></tr>
<tr><td>Orbital altitude</td><td colspan="2">SLCCI</td></tr>
<tr><td>Range</td><td colspan="2">Ku-band</td></tr>
<tr><td>Ionosphere</td><td>Smoothed dual-frequency</td><td>GIM/NIC09</td></tr>
<tr><td>Wet troposphere</td><td colspan="2">Radiometer</td></tr>
<tr><td>Dry troposphere</td><td colspan="2">ERA</td></tr>
<tr><td>Dynamic atmosphere</td><td colspan="2">MOG2D*</td></tr>
<tr><td>Ocean tide</td><td colspan="2">GOT4.10*</td></tr>
<tr><td>Loading tide</td><td colspan="2">GOT4.10*</td></tr>
<tr><td>Solid Earth tide</td><td colspan="2">Cartwright*</td></tr>
<tr><td>Pole tide</td><td colspan="2">Desai2015**</td></tr>
<tr><td>Sea state bias</td><td>Gaspar/CSR/CLS</td><td>Gaspar</td></tr>
<tr><td>Mean sea surface</td><td colspan="2">DTU15</td></tr>
</table>

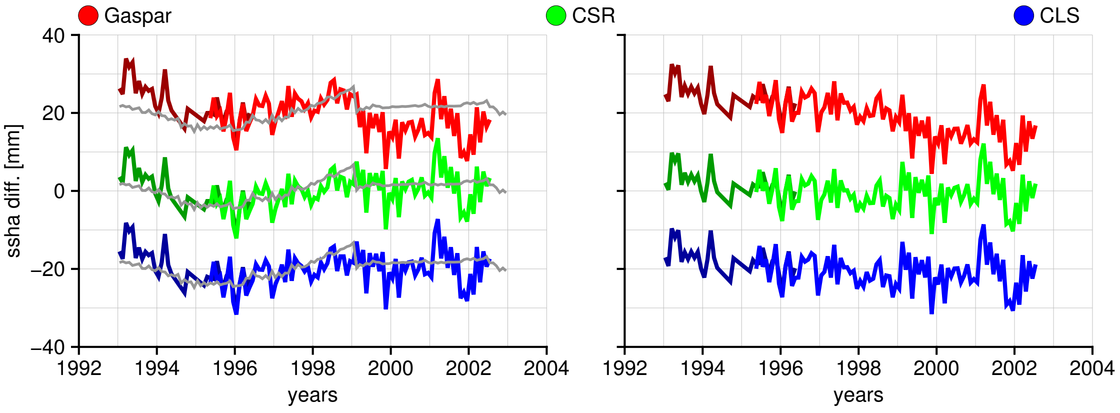

Figure 1: Time series of crossovers differences between TOPEX and ERS1&2. In the left panel the time series with cal-1 applied and on the right with cal-1 removed.

After removing the internal path delay calibration (cal-1) the time series becomes more linear (Fig. 1, right panel). It reduces the discontinuity between

TOPEX-A&B for the Gaspar SSB correction, but it induces an offset for the other two solutions. Note that TOPEX sea surface heights are negatively drifting with respect to those of ERS-1&2.

To estimate these drifts and intramission biases, we fit three models to the time series: 1) one TOPEX-A/B drift, 2) one TOPEX-A/B drift and an intramission bias and 3) two separate TOPEX-A&B drifts and an intramission bias. The statistical significance of an improvement of models 2 and 3 with respect to model 1 determined with an F-test:

$$F = \frac{(e_1^T e_1 - e_i^T e_i)/g}{e_i^T e_i/(n-k)}, \tag{1}$$

where e_1 are the residuals after fitting model 1, e_i the residuals after fitting models 2 and 3, $(n-k)$ the degrees-of-freedom and g the number of additional estimated parameters with respect to model 1. As discussed in the main article, there is an improved fit for the CSR and CLS SSB corrections if model 2 is applied. The estimated drifts of TOPEX-A&B are statistically equal in model 3, independent of the SSB correction, so a single TOPEX-A/B drift estimate is sufficient. If model 2 is applied, the estimated drifts between TOPEX and ERS-1&2 are statistically equal for all three SSB corrections.

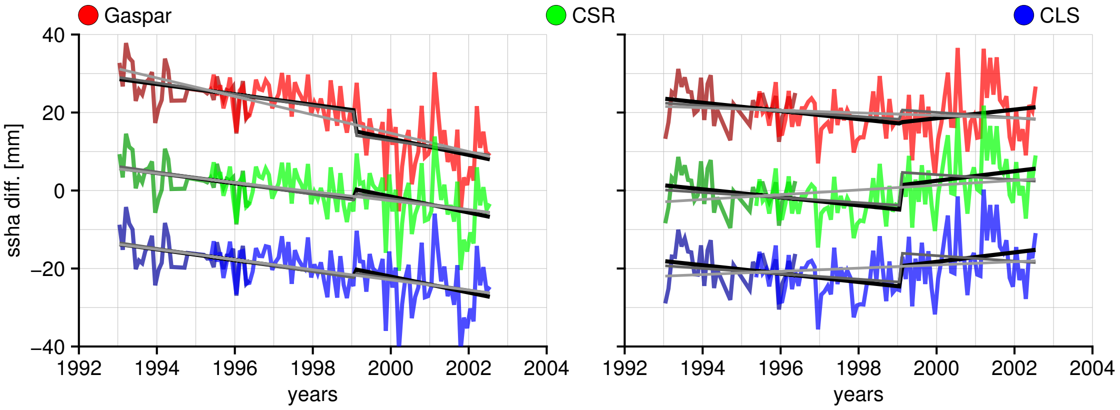

Figure 2: Time series of crossovers differences between TOPEX and ERS1&2 with cal-1 not applied. On the left the averaged time series for the southern hemisphere and on the right for the northern hemisphere.

We also investigate the geographical dependency of the drifts and the intramission biases. Fig. 2 shows the crossover time series southern and northern hemispheres and Table 2 contains the drifts and intramission biases with statistics. The cause for these geographical differences is unknown, but it is likely related to the orbit solution. For the southern hemisphere negative drifts are obtained

for TOPEX-A&B, while for the northern hemisphere the TOPEX-B drift is positive in model 3. Globally, as shown in Fig. 1 of the main article, the drifts for TOPEX-A&B are both negative. The intramission biases computed for the CLS and CSR SSB corrections over the southern hemisphere are negligible, while globally these are significantly different from zero. Between the northern and southern hemisphere there is a substantial difference between the estimated intramission biases. Note that on the northern hemisphere the choice of model (2 or 3) affects the magnitude of the estimated intramission bias. The geographical dependency of the intramission bias and the TOPEX-B drift implies that a calibration is only valid for Global Mean Sea Level (GMSL) and is not directly applicable in regional studies.

Table 2: Estimated drifts and biases from crossovers of TOPEX-ERS1&2 for both hemispheres.

SSB	Cal-1	Model	Drift A [mm yr^{-1}]	Drift B [mm yr^{-1}]	Bias A/B [mm]
			Southern hemisphere		
Gaspar	no	1	-2.36±0.19		-
CSR	no	1	-1.17±0.19		-
CLS	no	1	-1.30±0.19		-
Gaspar	no	2	-1.46±0.34		-6.1±1.9
CSR	no	2	-1.43±0.34		1.7±1.9
CLS	no	2	-1.48±0.34		1.2±1.9
Gaspar	no	3	-1.33±0.37	-2.08±0.84	-5.3±2.1
CSR	no	3	-1.30±0.37	-2.04±0.84	2.5±2.1
CLS	no	3	-1.37±0.37	-2.04±0.83	1.9±2.1
			Northern hemisphere		
Gaspar	no	1	-0.33±0.20		-
CSR	no	1	0.61±0.20		-
CLS	no	1	0.41±0.21		-
Gaspar	no	2	-0.69±0.37		2.4±2.1
CSR	no	2	-0.65±0.35		8.5±2.0
CLS	no	2	-0.69±0.37		7.4±2.1
Gaspar	no	3	-1.05±0.39	1.12±0.88	0.3±2.2
CSR	no	3	-1.03±0.37	1.23±0.83	6.3±2.1
CLS	no	3	-1.07±0.39	1.21±0.88	5.2±2.2

3 Altimeter-tide gauge processing

For the comparison with tide gauges, we use a procedure slightly modified with respect to *Watson et al.*[4]. Instead of the fast delivery products (fast), the hourly research quality tide-gauge records (rqds) from UHSLC[5] are used. Initially, all records that span the period 1993.0-2002.5 are considered. The tide-gauge records

within a radius of 1000 km from ≥7.5 moment-magnitude earthquake events during that period are removed.

All altimetry time series within a radius of 220 km around a tide gauge are taken into account, with a minimum distance from the coast of 30 km to avoid land signals contaminating waveforms and radiometer wet troposphere delay estimates. The TOPEX data are colinearly stacked, so that altimetry time series are created at every 6 km along-track. ALT-TG sea-level differences larger than 1 meter are removed.

Linear corrections for Vertical Land Motion (VLM) are estimated from models or from GNSS trends. We take the median of all GNSS trends within 50 km from the tide gauge in the database of the Nevada Geodetic Laboratory (NGL)[6]. Only GNSS trends with a formal uncertainty smaller than 1 mm yr^{-1} are included. To cope with VLM differences between the time spans of GNSS and TOPEX, due to non-linear present-day mass redistribution, we use the VLM estimates based on the models and loads used by *Frederikse et al.*[7]. The correction is implemented as in *Kleinherenbrink et al.*[8] and implicitly also deals with the problem of polar wander, which is not captured by the IERS2010 pole tide used as background model for GNSS and altimetry. Note that *Kleinherenbrink et al.*[8] showed that the consistency between altimetry, tide-gauges and GNSS trends improved if the non-linear VLM is taken into account. In case there are no GNSS stations nearby the tide gauge, the linear trends from the present-day mass redistribution VLM model and the ICE-6G_C VM5a GIA model[9] are used to estimate the total VLM trend at the tide gauge. That means that for tide gauges without a nearby GNSS receiver, a part of the (primarily small-scale) VLM variations cannot be taken into account, but it increases the number of tide gauge that can be used for validation.

From every altimetry time series, cubicly interpolated UHSLC tide gauge sea level measurements are subtracted, creating an ALT-TG differenced time series at every 6 km along-track. Closely following *Watson et al.*[4], a model is regressed through the ALT-TG time series containing 12 ocean tides, a latitude and longitude dependence complemented with altimeters drifts and intramission biases as model 1-3 in Sect. 2. Spectrograms of the residuals are computed and harmonics corresponding to peaks larger than $4\sigma^2$ are added to the model after which it is recomputed. Outliers outside 3RMS of the residuals are iteratively removed. Drifts and intramission bias estimates are only considered if their corresponding ALT-TG time series still contain at least 250 samples at this stage.

Thresholds are set to remove noisy Control Point (CP) time series, which require the propagation of uncertainties. To estimate the uncertainties of trends and biases, which are computed from the CP time series using ordinary least-squares, we fit an AR(1)-model through the residuals. With that we construct

variance-covariance matrix Q_{yy}:

$$Q_{yy} = \begin{pmatrix} \sigma^2 & \phi_1\sigma^2 & \cdots & \phi_1^n\sigma^2 \\ \phi_1\sigma^2 & \sigma^2 & \cdots & \phi_1^{n-1}\sigma^2 \\ \vdots & \vdots & \ddots & \vdots \\ \phi_1^n\sigma^2 & \phi_1^{n-1}\sigma^2 & \cdots & \sigma^2 \end{pmatrix}, \tag{2}$$

where σ is the standard deviation of the residuals and ϕ_1 the first-lag autocorrelation. By propagation of errors, we estimate the variance-covariance matrix for the estimated parameters as:

$$Q_{xx} = (A^T Q_{yy}^{-1} A)^{-1}, \tag{3}$$

where A is the design matrix used in the ordinary least-squares estimate described above. The standard deviations for the drifts and biases are extracted from the matrix Q_{xx}. We remove the time series for which the standard deviations of the residuals is larger than 110 mm, with a TOPEX-A drift uncertainty larger than 10 mm yr^{-1} (both as in *Watson et al.*[4]) or a TOPEX-A/B drift uncertainty larger than 8 mm yr^{-1}.

To investigate the stability of the ALT-TG comparison, the drifts and intramission biases are averaged using four methods. First the unweighted average trends and biases (m1) at every along-track location. The mean and uncertainties of the drifts and biases are computed from distributions estimated with a Monte-Carlo simulation, in which we randomly leave twenty tide gauges out of consideration. These uncertainties only reflect changes due to weighting and network geometry. The uncertainties are inflated by 0.3 mm yr^{-1} to account for reference frame stability issues[11] and differences between long-term ALT-TG and GNSS VLM trends[8]. This is done for all four methods. The second method is similar to *Watson et al.*[4] and weights the trends and biases with their variance (m2). A 1.5 mm yr^{-1} uncertainty for the GNSS trends is taken into account. Third, the means of trends and biases are computed for every tide gauge and consecutively all mean trends and biases are averaged (m3). Only tide gauges that are coupled with at least ten trends/biases are included, which makes the method less prone to outliers. Finally, a virtual-station method[12] is applied (m4). This method takes an average of the trends and biases between the nearest tide gauges and creates a virtual station at the midpoint. The virtual station is added and the two original stations are removed. This process loops until no stations are closer than 500 km from each other, which should be enough to remove most correlations between neighbouring stations and which creates a more homogeneously spaced network. The limit of 500 km is chosen to avoid generation of high weights for several remote stations, which would make the result sensitive to outliers. A second consideration for this radius is that in most regions ocean signals correlate at smaller radii[13]. Similar to the third method, a minimum of ten trends and biases is required per tide gauge.

4 Tide-gauge comparison

A tide-gauge comparison is required to validate the results of the crossovers, i.e. it is required to determine whether the TOPEX sea surface heights are drifting or the ERS1&2 sea surface heights. To verify whether a tide-gauge comparison is suitable to estimate such short-term drifts, the network geometry, the data editing and the distribution of the Monte Carlo simulations are investigated.

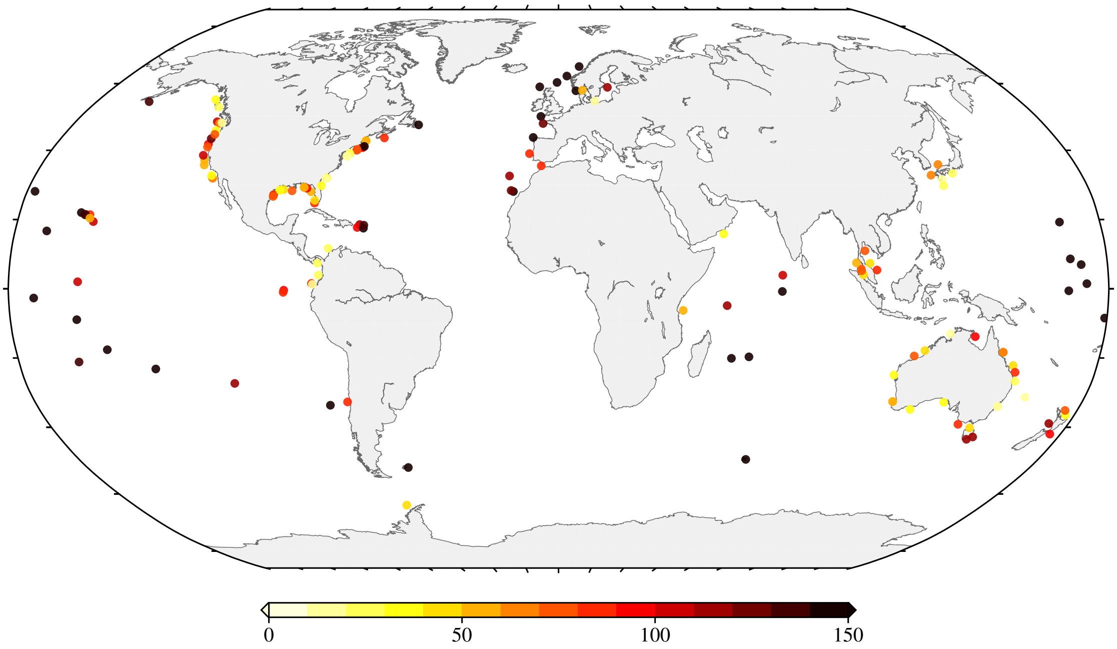

Figure 3: Number of CPs per tide gauge used for the results of model 1 based on the Gaspar SSB correction. Only stations with more than ten CPs are shown.

Geographical distribution

The number of ALT-TG time series and the corresponding drifts and intramission biases varies slightly, depending on the model (1-3) and the applied SSB correction. For the Gaspar SSB correction in combination with fitting model 1, the number ALT-TG solutions per tide gauge is given in Fig. 3. In general, higher numbers are found near island-based tide gauges. This is mainly because altimetry data are available from all sides of the island. Additionally, the ocean signals over continental shelves have a short correlation distance[14] and therefore residual they can show up in ALT-TG time series. A typical example of this is given in *Kleinherenbrink et al.*[8], where ENSO-related signals are visible at the Winter Harbour tide-gauge in Canada. These residual ocean signals inflate the estimated drift and bias uncertainties and as a consequence a part of the ALT-TG time drifts and biases are

removed from the analysis. The (correlated) residuals enhance the uncertainties of the drifts, so that part of them will be removed due to the thresholds set in Sect. 3. At several high-latitude locations the number of ALT-TG time series also increases due to the smaller ground-track spacing towards the poles.

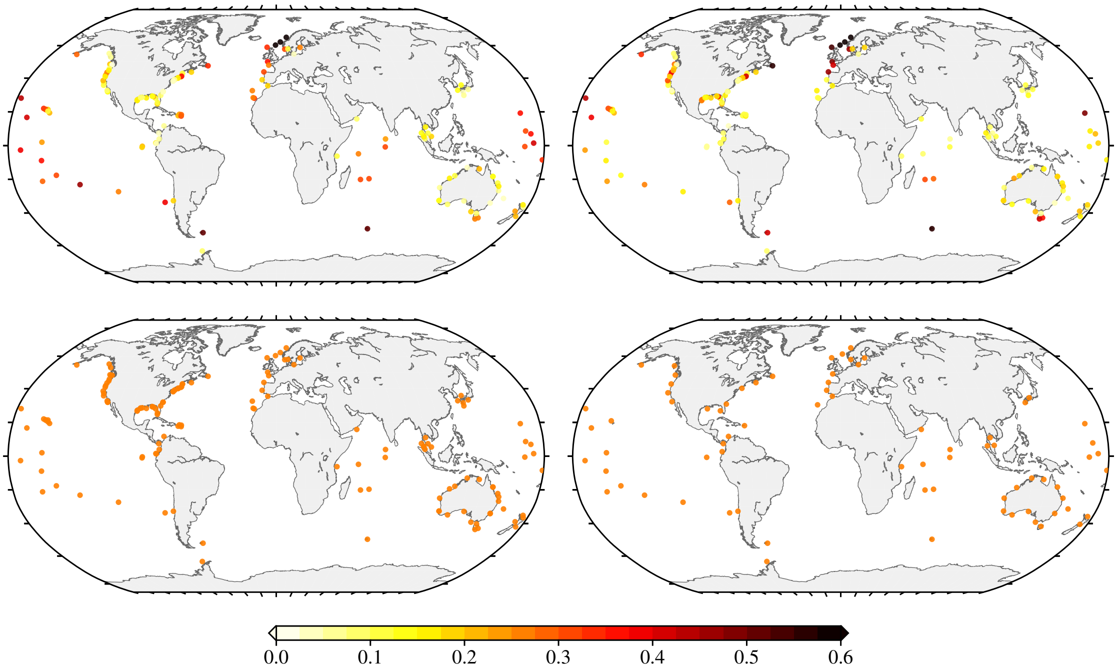

Figure 4: Relative weight per tide gauge or virtual station. Top-left: m1 (mean). Top-right: m2 (variance weighting). Bottom-left: m3 (mean per station). Bottom-right: m4 (virtual stations).

The varying number of ALT-TG solutions per tide gauge directly translates into under- and overweighting of certain regions in a tide-gauge-based satellite altimetry validation and/or calibration procedure. For the four weighting methods (m1-m4) the relative weight per tide gauge is shown in Fig. 4. Method m1, the unweighted mean, strongly weights drifts and intramission biases estimated at tide-gauges in Europe and the Pacific and Indian Ocean islands. In method m2, the variance weighting, a similar pattern emerges, but the weights for the island-based tide gauges in the Pacific is reduced. Both aforementoined methods have relatively low weights for the tide-gauge at the North-American coasts, but note that there are many tide gauges in this region. Due to the density of tide gauges at the North-American coasts, a relatively large weight is given to this region by the tide-gauge average method m3. The virtual-station method m4 improves the homogeneity of the stations (bottom-right panel of Fig. 4). However, all methods suffer from undersampling of certain regions. Especially the soutern oceans lack

tide gauges.

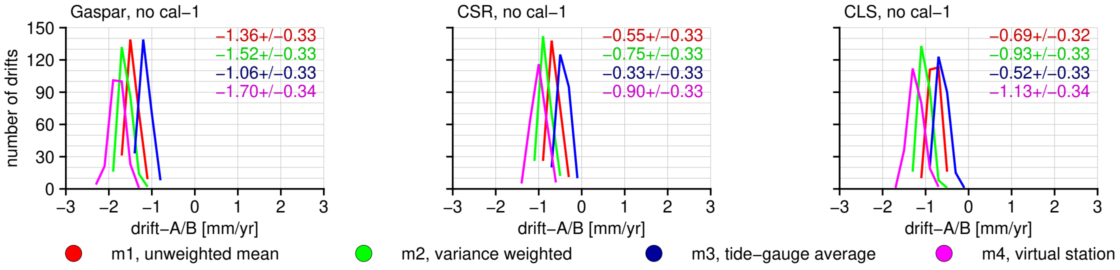

Figure 5: Histograms of the TOPEX A/B drift for the four averaging methods based on model 1 without cal-1 applied to TOPEX-A&B. On the left, at the middle and on the right the histograms for the Gaspar, CSR and CLS SSB corrections. In red, green, blue and purple the methods m1, m2, m3 and m4, respectively.

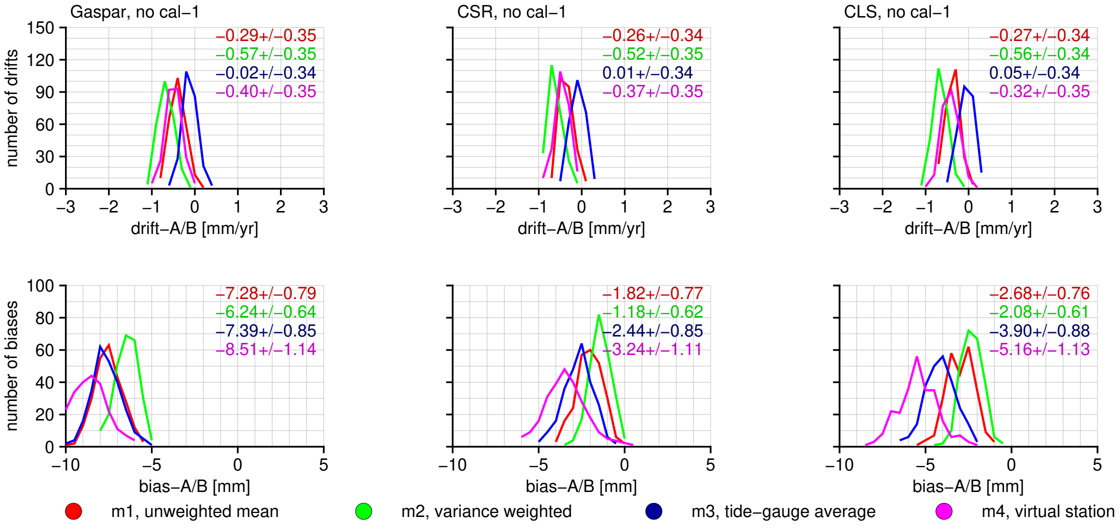

Figure 6: Histograms of the TOPEX A/B drift and intramission bias for the four averaging methods based on model 2 without cal-1 applied to TOPEX-A&B. On the left, at the middle and on the right the histograms for the Gaspar, CSR and CLS SSB corrections. In red, green, blue and purple the methods m1, m2, m3 and m4, respectively.

Histograms

We inspect the stability of the tide-gauge comparison using histograms for the three models. The results are considered to be insensitive if statistically consistent drifts and intramission biases are obtained using methods (m1-4) in combinations

with the regressed models. As a reference, the three models are computed through the time series with the cal-1 correction unapplied. The histograms of the drifts and biases estimated with models 1 (Fig. 7), model 2 (Fig. 6) and model 3 (Fig. 5) demonstrate the effects of the weighting methods. With model 1, a drift of slightly larger magnitude is found for the Gaspar SSB time series than for the other two, which is consistent with the crossovers (main text, Table 1). Since the drifts for model 1 are statistically consistent with the crossover drifts, it suggests that TOPEX sea surface heights are drifting and not the ERS1&2 sea surface heights.

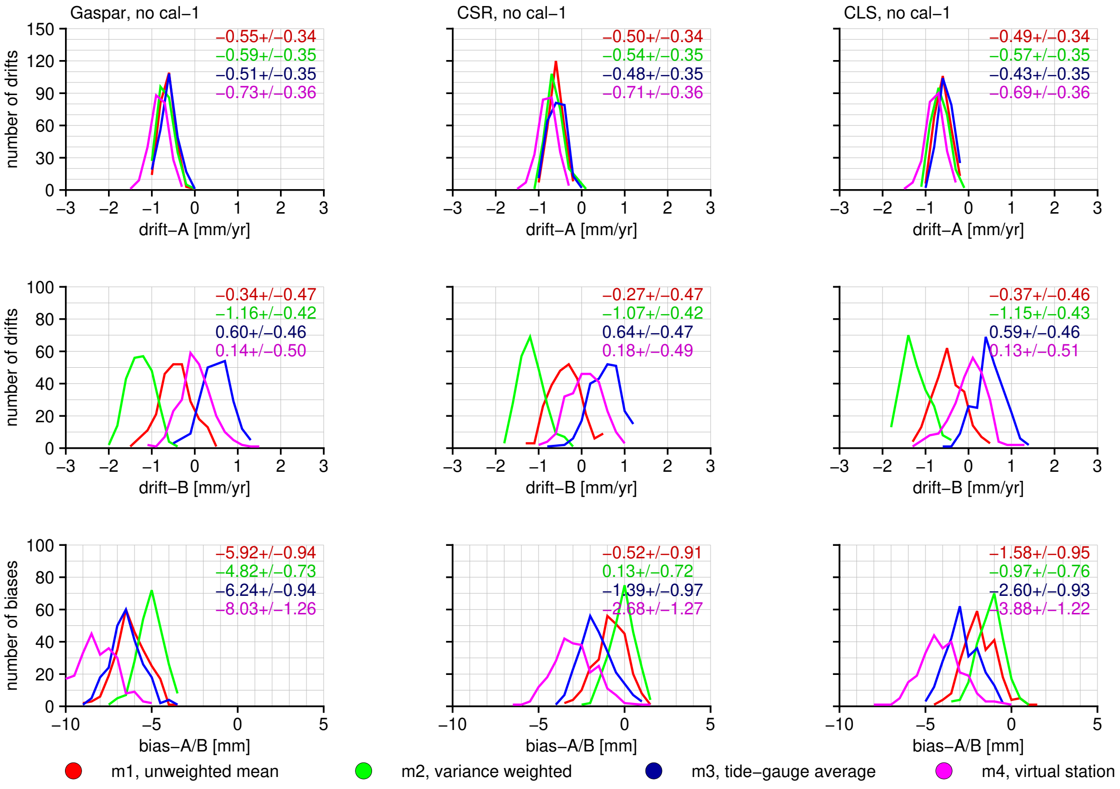

Figure 7: Histograms of the TOPEX A&B drifts and intramission bias for the four averaging methods based on model 3 without cal-1 applied to TOPEX-A&B. On the left, at the middle and on the right the histograms for the Gaspar, CSR and CLS SSB corrections. In red, green, blue and purple the methods m1, m2, m3 and m4, respectively.

However, when additionally an intramission bias (model 2) is estimated in the ALT-TG time series, different results are obtained than for the crossovers (Fig. 6). Independent of the averaging method and the SSB correction the intramission biases become 4-6 mm lower than those found when using the crossovers. This

leads to TOPEX-A/B drifts that are statistically indistinguishable from zero. Also note that for the methods with the largest drift, the smallest intramission bias is found and vice versa. A possible source for this is the geographic dependence of the intramission bias, as demonstrated with the crossovers. The geographically varying intramission bias in combination with the over- and underweighting of certain regions in the tide-gauge comparison affects the average result. We argue that the intramission bias obtained from the crossovers is therefore more accurate. Residual ocean signals in ALT-TG time series[15], which correlate between tide gauges, are another source for the difference in results. ALT-TG bias estimates from CPs located in the tropical Pacific and along the American shore are especially prone to remaining signals from the consecutive 97-98 El Niño[8] and 99-00 La Niña events, because they occurr around the time of the TOPEX-A/B transition.

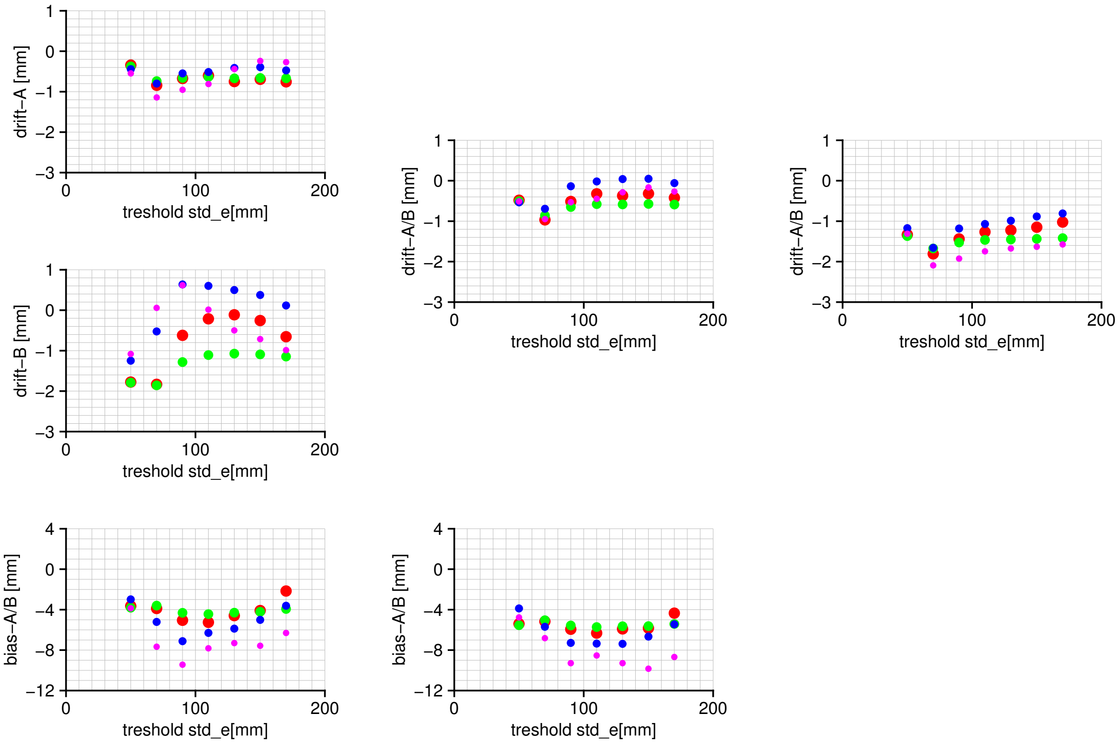

Figure 8: Test for sensitivity of the estimated drifts and biases to the threshold on the standard deviations of the residuals. Left: two separate drifts are estimated for TOPEX-A&B and and intramission bias (model 3). Middle: one drift is estimated for TOPEX-A/B and an intramission bias (model 2). Right: only one drift for TOPEX-A/B is estimated. The methods m1, m2, m3 and m4 are plotted in red, green, blue and pink, respectively.

The statistics of model 3 (Fig. 7) show that the averaging methods find large

differences in the drift of TOPEX-B. The estimated biases are closer to zero with respect to model 2 and appear to be negatively correlated with the drifts in TOPEX-B. The drifts in TOPEX-A are negative for methods m1, m2 and m3 and slightly higher for m4, but all of them close to zero and statistically consistent between the methods. Large deviations between the methods suggest that geographically varying signals affect the global estimate. Additionally, for estimating an accurate drift through TOPEX-B, the time series appear is too short. Since the intramission bias is geographically varying and both drifts and the intramission biases are susceptible to correlating ocean signals from for example ENSO, we recommend to use model 1 and not the other two for validation purposes.

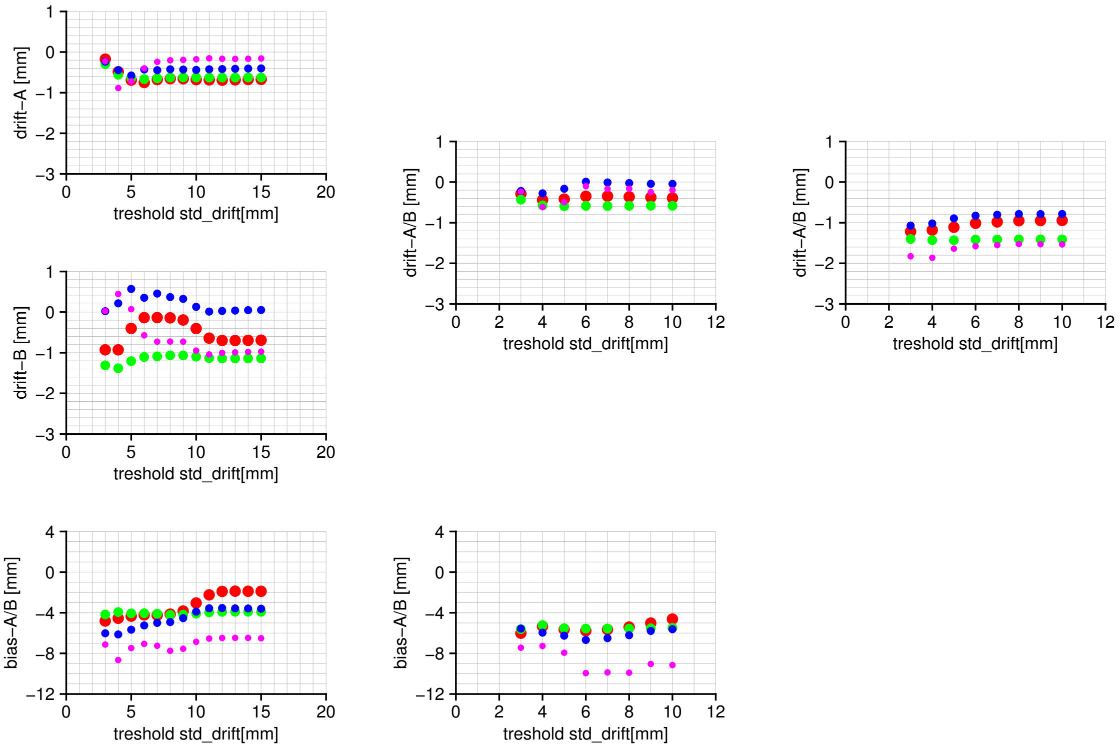

Figure 9: Test for sensitivity of the estimated drifts and biases to the threshold on the standard deviations of the TOPEX-A and TOPEX-A/B drifts. Left: two separate drifts are estimated for TOPEX-A&B and and intramission bias (model 3). Middle: one drift is estimated for TOPEX-A/B and an intramission bias (model 2). Right: only one drift for TOPEX-A/B is estimated. The methods m1, m2, m3 and m4 are plotted in red, green, blue and pink, respectively.

Thresholds
Preferably, the estimated drifts and intramission biases do not vary significantly between averaging methods (m1-4) and when thresholds are varied. To determine the effect of the thresholds used in the ALT-TG analysis on the model parameters, estimates of drifts and intermission biases are made at varying thresholds (Fig. 8 and Fig. 9). In Fig. 8, the threshold on the standard deviation of the residuals is varied while we set the trend uncertainty thresholds to 100 mm yr^{-1}. In Fig. 9, the threshold on the trend uncertainty is varied, while the threshold on the standard deviation of the residuals is set to 200 mm. Note that the nominal thresholds were 110 mm for the standard deviation and 10 mm yr^{-1} and 8 mm yr^{-1} for the trend uncertainties of TOPEX-A and TOPEX-A/B, respectively. The middle right panels show that it is difficult to constrain a drift for TOPEX-B, which supports the findings in the histograms. The TOPEX-B drifts between methods differ more than 1 mm yr^{-1} and the estimated drift strongly depends on the threshold for the standard deviation of residuals. This also appears to negatively correlate with the estimated intramission bias and slightly with the estimated TOPEX-A drift. As the results are unstable, we do not recommend to fit a model with two separate drifts for TOPEX-A&B for validation purposes, and certainly not for calibration.

More stable results are obtained for models with a single drift for TOPEX-A/B. The virtual station method (m4) yields a larger intramission bias at higher thresholds than the other methods. This is possibly related to the overall geometry of the network, but the method is also more prone to outliers as shown by the wider distributions in the histograms. The estimated TOPEX-A/B drift in both models for all methods is statistically consistent between methods. In conclusion, since statistically consistent results are only obtained by model 1, we recommend to use only model 1 for validations purposes.

References

[1] Scharroo, R. et al. RADS: Consistent multi-mission products. In Proceedings of Symposium on 20 Years of Progress in Radar Altimetry, 20, (2012).

[2] Desai, S., Wahr, J., & Beckley, B. Revisiting the pole tide for and from satellite altimetry. Journal of Geodesy, 89(12), 1233-1243 (2015).

[3] Petit, G., & Luzum, B. IERS conventions (2010) (No. IERS-TN-36). Bureau International Des Poids Et Mesures Sevres, France, ISBN 3-89888-989-6 (2010).

[4] Watson, C. S. et al. Unabated global mean sea-level rise over the satellite altimeter era. Nature Climate Change, 5(6), 565-568 (2015).

[5] Caldwell, P. C., Merrifield, M. A., & Thompson, P. R. Sea level measured by tide gauges from global oceans - the Joint Archive for Sea Level holdings (NCEI Accession 0019568). Version 5.5, NOAA National Centers for Environmental Information; 10.7289/V5V40S7W (2015).

[6] Blewitt, G., Kreemer, C., Hammond, W. C., & Gazeaux, J. MIDAS robust trend estimator for accurate GPS station velocities without step detection, Journal of Geophysical Research: Solid Earth, 121, 2054-2068 (2016).

[7] Frederikse, T. et al. Closing the sea level budget on a regional scale: Trends and variability on the Northwestern European continental shelf. Geophysical Research Letters, 43(20); 10.1002/2016GL070750 (2016).

[8] Kleinherenbrink, M., Riva, R., & Frederikse, T. A comparison of methods to estimate vertical land motion trends from GNSS and altimetry at tide gauge stations. Ocean Science, 14(2), 187-204 (2018).

[9] Peltier, W.R., Argus, D.F., & Drummond, R. Space geodesy constrains ice-age terminal deglaciation: The global ICE-6G_C model. J. Geophys. Res. Solid Earth, 120, 450-487 (2015).

[10] Rudenko, S., Schne, T., Neumayer, K. H., Esselborn, S., Raimondo, J. C., & Dettmering, D. GFZ VER11 SLCCI precise orbits of altimetry satellites ERS-1, ERS-2, Envisat, TOPEX/Poseidon, Jason-1 and Jason-2 in the ITRF2008; 10.5880/GFZ.1.2.2018.001 (2016).

[11] Santamaría-Gómez, A. et al. Uncertainty of the 20th century sea-level rise due to vertical land motion errors. Earth and Planetary Science Letters, 473, 24-32 (2017).

[12] Jevrejeva, S., Grinsted, A., Moore, J. C., & Holgate, S. Nonlinear trends and multiyear cycles in sea level records. J. Geophys. Res., 111; 10.1029/2005JC003229 (2006).

[13] Ducet, N., Le Traon, P. Y., & Reverdin, G. Global high-resolution mapping of ocean circulation from TOPEX/Poseidon and ERS-1 and -2. Journal of Geophysical Research: Oceans, 105(C8), 19477-19498 (2000).

[14] Hughes, C.W., & Meredith, M.P. Coherent sea-level fluctuations along the global continental slope. Phil. Trans. R. Soc., 364, 885-901 (2006).

[15] Vinogradov, S. V., & Ponte, R. M. Low-frequency variability in coastal sea level from tide gauges and altimetry. Journal of Geophysical Research: Oceans, 116(C7); 10.1029/2011JC007034 (2011).
